# Prevalence and Factors Associated With Nocturia (≥ 2 Night‐Time Urinary Episodes) in Japan: Analysis of the 2023 Japan Community Health Survey (JaCS 2023)

**DOI:** 10.1111/iju.70504

**Published:** 2026-05-15

**Authors:** Satoru Kira, Hiroshi Shimura, Takanori Mochizuki, Norifumi Sawada, Noritoshi Sekido, Naoya Masumori, Yoshiyuki Kojima, Takahiko Mitsui

**Affiliations:** ^1^ Department of Urology Interdisciplinary Graduate School of Medicine and Engineering University of Yamanashi Chuo Yamanashi Japan; ^2^ Department of Urology Toho University Ohashi Medical Center Meguro Japan; ^3^ Department of Urology Sapporo Medical University School of Medicine Sapporo Japan; ^4^ Department of Urology Fukushima Medical University School of Medicine Fukushima Japan

**Keywords:** epidemiological survey, lower urinary tract symptoms, nocturia

## Abstract

**Objectives:**

To investigate the prevalence and factors associated with nocturia, defined as ≥ 2 times/night, among participants from the 2023 Japan Community Health Survey (JaCS 2023).

**Methods:**

Using data from JaCS 2023, a nationwide online survey comprising 48 questionnaire items on lower urinary tract symptoms and daily life, 6210 participants aged 20–99 years were evaluated. Based on the definition of nocturia, participants were divided into Nocturia and Non‐nocturia groups. The prevalence of nocturia was examined, and multivariable logistic regression analysis was performed to explore factors associated with nocturia by sex. In addition, subgroup analyses were performed for participants aged < 60 and ≥ 60 years.

**Results:**

The overall prevalence of nocturia was 27.1% in men (aged ≥ 40 years: 31.4%, aged ≥ 60 years: 39.8%, aged ≥ 80 years: 58.1%) and 17.8% in women (aged ≥ 40 years: 19.9%, aged ≥ 60 years: 24.7%, aged ≥ 80 years: 38.2%) and increased with age (*p* for trend < 0.0001). Age, performance status (PS) ≥ 1, hypertension, depression/anxiety, insomnia, benign prostatic hyperplasia (BPH), fecal incontinence, alcohol intake more than once a week, and erectile dysfunction (ED) in men, and age, PS ≥ 1, decreased walking speed, hypertension, and insomnia in women were independently associated with nocturia. Subgroup analysis showed that ED in all male groups and PS ≥ 1 in all female groups were significant factors.

**Conclusions:**

The prevalence of nocturia increases with age. Age, PS ≥ 1, insomnia, and hypertension were significantly associated with nocturia in both men and women.

**Abbreviations**
BMI: body mass index
BPH: benign prostatic hyperplasia
ED: erectile dysfunction
HL: hyperlipidemia
HT: hypertension
JaCS 2023: the 2023 Japan Community Health Survey
LUTS: lower urinary tract symptoms
OAB: overactive bladder
POP: pelvic organ prolapse
PS: Performance Status
SAS: sleep apnea syndrome

## 1 Introduction

Nocturia is one of the most bothersome and common lower urinary tract symptoms (LUTS). Although the International Continence Society defines nocturia as the need to void one or more times during the night, experiencing two or more night‐time urinary episodes has been associated with deterioration in quality of life and an increased risk of mortality [1, 2, 3]. The major etiologies of nocturia include reduced bladder capacity, large production of urine, and sleep disorders, occurring either individually or in combination. These etiologies are associated with numerous factors such as lifestyle behavior, past and/or present medical history, general health condition, activities of daily living, and comorbid diseases. Therefore, epidemiological study is considered essential for elucidating factors associated with nocturia.

In Japan, a nationwide epidemiological survey on LUTS was conducted in 2002 [4, 5]. Since then, Japan has been facing a super‐aging society more rapidly than the other developed countries. To clarify the current prevalence of LUTS and the impact on daily life, the 2023 Japan Community Health Survey (JaCS 2023), a nationwide online epidemiological survey, was conducted by the Japanese Continence Society in 2023 [6]. The findings showed that nocturia had the greatest impact on daily life and was the most distressing symptom among both men and women in all age groups [6]. Numerous factors associated with nocturia have been reported in epidemiological studies conducted across various countries or local areas. In the context of a rapidly super‐aging society, it is important to explore the latest factors associated with nocturia; however, these are unknown.

Therefore, the aim of this study was to clarify the prevalence of nocturia and to identify factors associated with nocturia using data from JaCS 2023.

## 2 Methods

### 2.1 Participants

Participant selection and methodology for this epidemiological study have been reported previously [6]. Concisely, an online survey consisting of 48 questions was conducted among populations anonymously registered with the web panel of a Japanese research company (Macromill Inc., Tokyo, Japan) between May 31 and June 5, 2023. All participants, comprising 3122 men and 3088 women aged 20–99 years, were selected through probability sampling and quota‐based allocation designed to match the composition of the Japanese population according to the 2020 National Census of the Statistics Bureau, Ministry of Internal Affairs and Communications (https://www.stat.go.jp/english/data/kokusei/2020/summary.html). To ensure representativeness, participants were sampled to align with the census distribution for age, sex, and region; therefore, post‐stratification weights were not applied to the prevalence estimates or regression models.

All participants provided consent through an opt‐in method, and this study was approved by the Nihon University Itabashi Hospital Clinical Research Judging Committee (ID 2023–04).

### 2.2 Questionnaire and Definition of Nocturia

The questionnaire used in this study has been described previously [6]. Briefly, it comprised items evaluating demographic characteristics, LUTS, and daily life during the past month. Nocturia was assessed using the following question: ‘During the past month, how many times did you typically wake up to urinate between the time you went to bed and the time you woke up in the morning?’. In this study, nocturia was defined as ≥ 2 night‐time urinary episodes because nocturia ≥ 2 night‐time was associated with deterioration of QOL [1]. Accordingly, all participants were categorized into Nocturia and Non‐nocturia groups by sex.

### 2.3 Potential Factors Associated With Nocturia

To identify potential factors associated with nocturia, the following items were assessed: age, body mass index (BMI), and several frailty‐related indicators, including Eastern Cooperative Oncology Group Performance Status (PS) ≥ 1, decreased walking speed, relatively poor or poor health condition, weight loss, falls, and infrequent exercise. These indicators were evaluated as individual risk factors to identify their specific associations with nocturia. Other clinical items included hypertension (HT), hyperlipidemia (HL), diabetes mellitus, heart failure, myocardial infarction, chronic kidney disease, stroke, spine or spinal cord disorders, neurological disease, depression/anxiety, sleep apnea syndrome (SAS), insomnia, fecal incontinence, constipation, alcohol intake more than once a week, parity (for women), menopause (for women), pelvic organ prolapse (POP) (for women), benign prostatic hyperplasia (BPH) (for men), and erectile dysfunction (ED) (for men).

### 2.4 Statistical Analysis

All statistical analyses were performed using JMP statistical software (JMP Pro, version 18.0.2; SAS Institute Inc., Cary, NC). Trends in the prevalence of nocturia across age categories and by sex were examined using the Cochran‐Armitage trend test. Furthermore, an interaction analysis between age and sex was performed using a multivariable logistic regression model to evaluate whether the impact of age on nocturia prevalence differed significantly by sex. Comparisons of demographic and clinical characteristics between the Nocturia and Non‐nocturia groups by sex were performed using the Mann–Whitney U test for continuous variables and the chi‐square test for categorical variables. Continuous variables were expressed as medians with interquartile ranges, and categorical variables as numbers (%).

To identify factors associated with nocturia, multivariable logistic regression analysis was performed by sex. Age was always included in the multivariable analysis, and other variables with a significance level < 0.05 in univariate analysis were included in the multivariable analysis. Subgroup analysis was additionally performed for those aged < 60 years and ≥ 60 years by sex using the same approach. This cutoff represents the midpoint of the age range in this study and aligns with the traditional retirement age in Japan. All *p*‐values were two‐sided, and *p* < 0.05 was considered statistically significant. Multicollinearity among the covariates was assessed using the variance inflation factor (VIF); a VIF < 5.0 was considered to indicate the absence of significant multicollinearity.

## 3 Results

### 3.1 Prevalence of Nocturia

**FIGURE 1:**
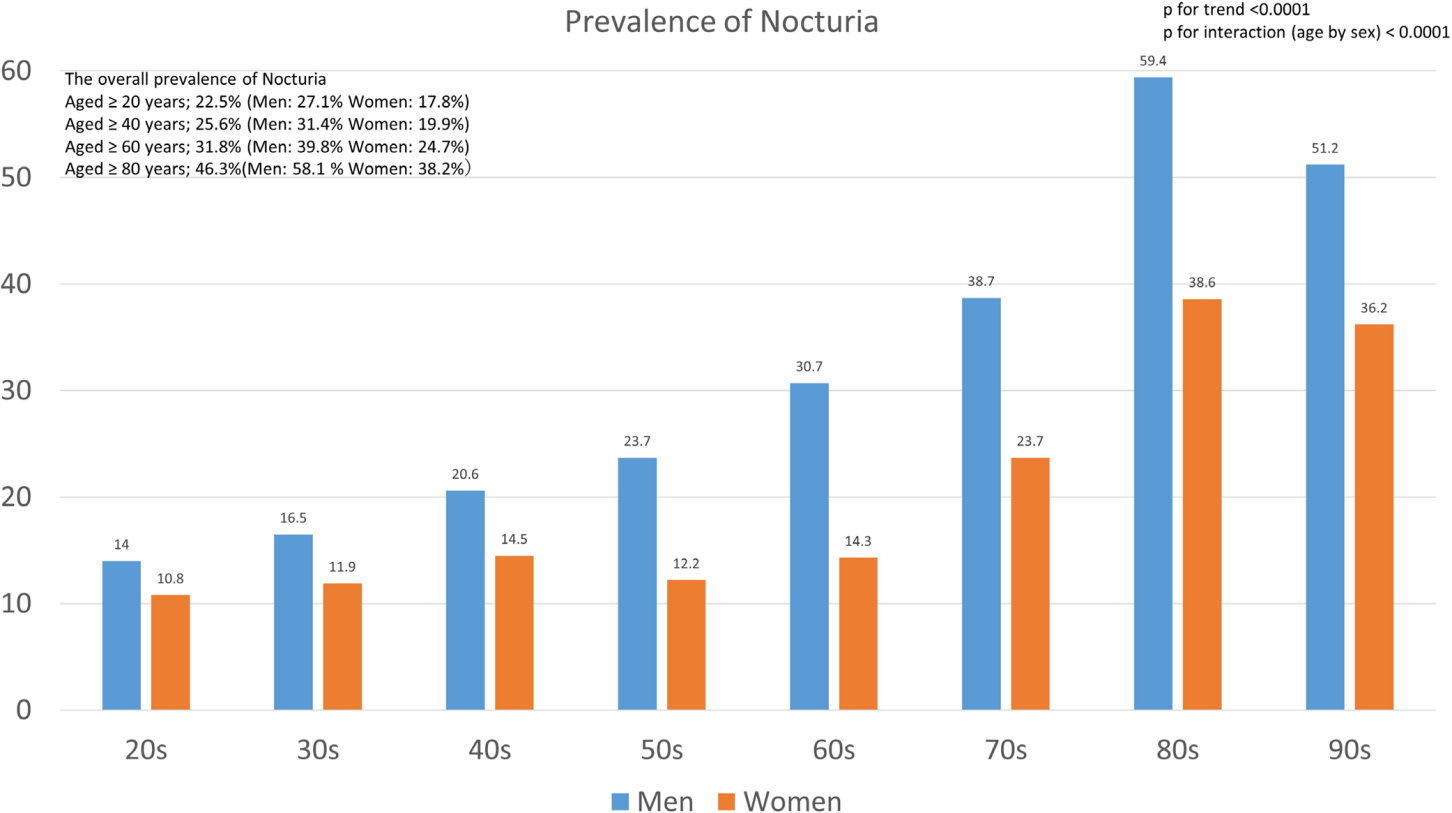
Prevalence of nocturia. The overall prevalence of nocturia was 27.1% in men (aged ≥ 40 years: 31.4%, aged ≥ 60 years: 39.8%, aged ≥ 80 years: 58.1%) and 17.8% in women (aged ≥ 40 years: 19.9%, aged ≥ 60 years: 24.7%, aged ≥ 80 years: 38.2%), and it increased with age. A significant age‐by‐sex interaction was observed (*p* < 0.0001).

The overall prevalence of nocturia is shown in Figure 1. The prevalence increased with age in both men and women (*p* for trend < 0.0001). It was 22.5% among participants aged ≥ 20 years (27.1% and 17.8% in men and women, respectively), 25.6% among those aged ≥ 40 years (31.4% and 19.9%), 31.8% among those aged ≥ 60 years (39.8% and 24.7%), and 46.3% among those aged ≥ 80 years (58.1% and 38.2%). When defined as ≥ 1 void per night according to the International Continence Society definition, the prevalence was 66.2% in men and 57.9% in women. In all age groups, the prevalence of nocturia was higher in men than in women. A significant age‐by‐sex interaction was observed (*p* < 0.0001), indicating that these sex differences became progressively more pronounced with advancing age (Figure 1).

### 3.2 Participants' Characteristics Between Nocturia Group and Non‐Nocturia Group

Comparisons of characteristics between the Nocturia and Non‐nocturia groups by sex are shown in Tables 1 and 2. Significant differences were observed for all items between groups except for weight loss in men, BMI, lack of exercise, and alcohol intake more than once a week in women.

**TABLE 1:** Participant characteristics between the Nocturia and non‐Nocturia groups in men.

	Non‐nocturia	Nocturia	*p*
*N*	2275	847	
Age, median (IQR)	49 (37–64)	63 (48–75)	< 0.0001
BMI, median (IQR)	22.8 (20.8–25.2)	23.0 (21.3–25.2)	0.0436
PS ≧ 1	254 (11.17)	215 (25.38)	< 0.0001
Relatively poor or poor health condition	246 (10.81)	162 (19.13)	< 0.0001
Weight loss	518 (22.77)	219 (25.86)	0.0719
Decreased walking speed	685 (30.11)	413 (48.76)	< 0.0001
Fall	432 (18.99)	229 (27.04)	< 0.0001
Lack of exercise	1025 (45.06)	342 (40.38)	0.0192
Hypertension	474 (20.84)	322 (38.02)	< 0.0001
Hyperlipidemia	246 (10.81)	137 (16.18)	< 0.0001
Diabetes mellitus	189 (8.31)	136 (16.06)	< 0.0001
Heart failure	45 (1.98)	41 (4.84)	< 0.0001
Myocardial infarction Myocardial Infarction	79 (3.47)	53 (6.26)	0.0006
Chronic kidney disease	46 (2.02)	40 (4.72)	< 0.0001
Stroke	56 (2.46)	39 (4.60)	0.0019
Spine/Spinal cord disorder	39 (1.71)	45 (5.31)	< 0.0001
Neurologic disease	31 (1.36)	30 (3.54)	< 0.0001
Depression/Anxiety	120 (5.28)	84 (9.92)	< 0.0001
Sleep apnea syndrome	79 (3.47)	73 (8.62)	< 0.0001
Insomnia	116 (5.1)	106 (12.52)	< 0.0001
BPH	103 (4.53)	131 (15.47)	< 0.0001
Fecal incontinence	29 (1.28)	56 (6.61)	< 0.0001
Constipation	146 (6.42)	124 (14.64)	< 0.0001
Alcohol intake	1094 (48.09)	496 (58.56)	< 0.0001
Erectile dysfunction	445 (19.56)	318 (44.66)	< 0.0001

Abbreviations: BMI, body mass index; BPH, benign prostatic hyperplasia; IQR, inter‐ quartile range; *N*, number; PS, performance status.

**TABLE 2:** Participant characteristics between the Nocturia and non‐Nocturia groups in women.

	Non‐nocturia	Nocturia	*p*
*N*	2537	551	
Age, median (IQR)	53 (39–69)	68 (47–80)	< 0.0001
BMI, median (IQR)	20.8 (19.0–23.1)	21.2 (19.1–23.8)	0.0543
PS ≧ 1	264 (10.41)	175 (31.76)	< 0.0001
Relatively poor or poor health condition	226 (8.91)	117 (21.23)	< 0.0001
Weight loss	498 (19.63)	131 (23.78)	0.0285
Decreased walking speed	863 (34.02)	304 (55.17)	< 0.0001
Fall	491 (19.35)	151 (27.41)	< 0.0001
Lack of exercise	1299 (51.20)	281 (51.00)	0.9308
Hypertension	374 (14.74)	168 (30.49)	< 0.0001
Hyperlipidemia	273 (10.76)	96 (17.42)	< 0.0001
Diabetes mellitus	98 (3.86)	44 (7.99)	< 0.0001
Heart failure	29 (1.14)	25 (4.54)	< 0.0001
Myocardial infarction	39 (1.54)	26 (4.72)	< 0.0001
Chronic kidney disease	35 (1.38)	22 (3.99)	< 0.0001
Stroke	36 (1.42)	22 (3.99)	< 0.0001
Spine/Spinal cord disorder	53 (2.09)	34 (6.17)	< 0.0001
Neurologic disease	31 (1.22)	15 (2.72)	0.0084
Depression/Anxiety	154 (6.07)	44 (7.99)	0.0962
Sleep apnea syndrome	38 (1.50)	19 (3.45)	0.002
Insomnia	158 (6.23)	72 (13.01)	< 0.0001
Fecal incontinence	36 (1.42)	35 (6.35)	< 0.0001
Constipation	331 (13.05)	103 (18.69)	0.0005
Alcohol intake	739 (29.13)	164 (29.76)	0.7569
Parity			0.0646
1 ~ 2	1227 (79.73)	285 (75.40)	
≧ 3	312 (20.27)	93 (24.60)	
Menopause	1086 (48.22)	280 (62.08)	< 0.0001
Pelvic organ prolapse	107 (4.51)	35 (7.01)	0.019

Abbreviations: BMI, body mass index; IQR, inter‐quartile range; *N*, number; PS, performance status.

### 3.3 Results of Multivariable Analysis

Tables 3 and 4 present the results of univariable and multivariable logistic regression analysis by sex. In all multivariable models, the VIF values for all included variables were less than 2.0, confirming the stability of the models. In men, age, PS ≥ 1, HT, depression/anxiety, insomnia, BPH, fecal incontinence, alcohol intake more than once a week, and ED were significantly associated with nocturia (Table 3). In women, age, PS ≥ 1, decreased walking speed, HT, and insomnia were significantly associated with nocturia (Table 4).

**TABLE 3:** Univariable and multivariable analyses for nocturia in men.

	Univariable analysis			Multivariable analysis		
	Odds ratio	95% CI	*p*	Odds ratio	95% CI	*p*
Age	1.035	1.030–1.040	< 0.0001	1.026	1.019–1.033	< 0.0001
BMI	1	0.986–1.011	0.9794			
PS ≧ 1	2.707	2.211–3.314	< 0.0001	1.452	1.104–1.910	0.0076
Relatively poor or poor health condition	1.951	1.571–2.422	< 0.0001	1.25	0.942–1.659	0.1222
Weight loss	1.183	0.986–1.420	0.0728			
Decreased walking speed	2.209	1.879–2.597	< 0.0001	1.082	0.873–1.340	0.4721
Fall	1.581	1.315–1.901	< 0.0001	1.199	0.956–1.505	0.1167
Lack of exercise	0.826	0.704–0.969	0.0193	0.828	0.683–1.005	0.0556
Hypertension	2.33	1.962–2.767	< 0.0001	1.284	1.027–1.606	0.0281
Hyperlipidemia	1.592	1.270–1.995	< 0.0001	0.895	0.678–1.183	0.4369
Diabetes mellitus	2.111	1.667–2.674	< 0.0001	1.021	0.756–1.379	0.8929
Heart failure	2.521	1.639–3.878	< 0.0001	0.957	0.518–1.769	0.8884
Myocardial infarction	1.855	1.298–2.652	0.0007	0.728	0.451–1.175	0.1941
Chronic kidney disease	2.402	1.560–3.697	< 0.0001	0.888	0.500–1.576	0.6847
Stroke	1.913	1.261–2.901	0.0023	0.637	0.364–1.115	0.1144
Spine/Spinal cord disorder	3.217	2.079–4.977	< 0.0001	1.244	0.688–2.248	0.4704
Neurologic disease	2.658	1.599–4.419	0.0002	0.918	0.420–2.006	0.8306
Depression/Anxiety	1.977	1.478–2.644	< 0.0001	1.603	1.083–2.373	0.0183
Sleep apnea syndrome	2.622	1.888–3.642	< 0.0001	1.458	0.956–2.223	0.08
Insomnia	2.662	2.020–3.510	< 0.0001	1.552	1.061–2.270	0.0235
BPH	3.858	2.941–5.062	< 0.0001	1.651	1.181–2.309	0.0034
Fecal incontinence	5.483	3.476–8.648	< 0.0001	2.3	1.289–4.101	0.0048
Constipation	2.501	1.940–3.223	< 0.0001	1.245	0.902–1.717	0.1821
Alcohol intake	1.525	1.301–1.789	< 0.0001	1.408	1.164–1.704	0.0113
Erectile dysfunction	2.666	2.223–3.198	< 0.0001	1.507	1.219–1.862	0.0002

Abbreviations: BMI, body mass index; BPH, benign prostatic hyperplasia; PS, performance status.

**TABLE 4:** Univariable and multivariable analyses for nocturia in women.

	Univariable analysis			Multivariable analysis		
	Odds ratio	95% CI	*p*	Odds ratio	95% CI	*p*
Age	1.029	1.023–1.034	< 0.0001	1.024	1.014–1.034	< 0.0001
BMI	1.017	1.003–1.038	0.0802			
PS ≧ 1	4.007	3.216–4.993	< 0.0001	2.015	1.472–2.756	< 0.0001
Relatively poor or poor health condition	2.757	2.156–3.524	< 0.0001	1.072	0.759–1.513	0.6933
Weight loss	1.277	1.026–1.590	0.0288	1.144	0.867–1.509	0.3406
Decreased walking speed	2.387	1.980–2.878	< 0.0001	1.575	1.237–2.007	0.0002
Fall	1.573	1.273–1.943	< 0.0001	1.079	0.828–1.405	0.574
Lack of exercise	0.992	0.825–1.193	0.931			
Hypertension	2.537	2.052–3.136	< 0.0001	1.392	1.038–1.866	0.0271
Hyperlipidemia	1.75	1.358–2.254	< 0.0001	0.913	0.655–1.273	0.5931
Diabetes mellitus	2.16	1.494–3.122	< 0.0001	0.929	0.550–1.567	0.7818
Heart failure	4.11	2.388–7.075	< 0.0001	2.102	0.811–5.451	0.1265
Myocardial infarction	3.172	1.914–5.256	< 0.0001	1.096	0.507–2.368	0.8158
Chronic kidney disease	2.973	1.730–5.109	< 0.0001	1.666	0.722–3.845	0.2313
Stroke	2.889	1.686–4.951	0.0001	1.087	0.470–2.514	0.845
Spine/Spinal cord disorder	3.082	1.983–4.790	< 0.0001	1.12	0.611–2.055	0.7134
Neurologic disease	2.262	1.213–4.220	0.0103	0.392	0.129–1.191	0.0985
Depression/Anxiety	1.343	0.9478–1.903	0.0973			
Sleep apnea syndrome	2.349	1.343–4.106	0.0027	0.764	0.318–1.836	0.5467
Insomnia	2.263	1.684–3.041	< 0.0001	1.903	1.292–2.801	0.0011
Fecal incontinence	4.712	2.931–7.576	< 0.0001	1.534	0.767–3.067	0.2264
Constipation	1.532	1.201–1.954	0.0006	1.042	0.766–1.418	0.791
Alcohol intake	1.031	0.843–1.261	0.7664			
Parity ≧ 3	1.283	0.985–1.673	0.0651			
Menopause	1.758	1.429–2.163	< 0.0001	0.705	0.497–1.002	0.0513
Pelvic organ prolapse	1.597	1.077–2.370	0.02	1.421	0.896–2.252	0.135

Abbreviations: BMI, body mass index; PS, performance status.

### 3.4 Results of Subgroup Analysis

Characteristics of each subgroup are shown in (Table S1) (Table S2). Among participants aged < 60 years, age, PS ≥ 1, HL, depression/anxiety, SAS, alcohol intake, and ED in men, and PS ≥ 1, weight loss, and insomnia in women were significant factors associated with nocturia (Tables S3 and S4) (Tables S5 and S6). Among participants aged ≥ 60 years old, age, HT, BPH, and ED in men, and age, PS ≥ 1, decreased walking speed, HT, fecal incontinence, and POP in women were significant factors (Tables S7 and S8).

## 4 Discussion

In this study, the prevalence of nocturia (≥ 2 night‐time urinary frequency) was 27.1% and 17.8% in men and women, respectively. Prevalence increased with age and was often higher in men than in women across all age groups. While these findings are similar to those of a survey conducted approximately 20 years ago, our study provides new insights into both ends of the age spectrum; it includes a notable sample of the oldest‐old in their 80s and 90s, while also revealing an interesting prevalence among both men and women in their 20s or 30s [4]. In contrast, several surveys from other countries have shown higher rates in women than in men [7, 8]. Differences in race, assessment parameters, methodology, or survey season may contribute to these discrepancies. However, the consistent prevalence of nocturia across previous surveys remains an important unresolved issue. From clinical and community perspectives, our findings highlight that a multi‐faceted approach, including physical functional assessment, may provide valuable insights for the future management of nocturia.

Multivariable analysis identified age, HT, insomnia, and PS ≥ 1 as independent factors associated with nocturia in both men and women. The associations with age and HT align with previous findings, and age has consistently been considered a strong factor associated with nocturia [9]. In a pathophysiological context, HT prevalence is considered higher in older people and might be associated with fluid retention and increased glomerular filtration rate, potentially linking it to nocturia as nocturnal polyuria. Our results further support the association between age, HT and nocturia.

It is known that nocturia can cause sleep disturbance, resulting in insomnia [10]. Furthermore, a longitudinal and population‐based health survey in a Japanese regional city showed a bidirectional correlation between nocturia and insomnia [11]. The association between insomnia and nocturia observed in the present study may reflect these findings.

In this study, frailty was not assessed using a formal clinical diagnostic definition. Instead, physical functional decline, a key component of frailty, was evaluated using the Performance Status (PS). Participants with PS ≥ 1 were considered to have decreased physical function as an indicator related to frailty. As confirmed by the low VIF values, these indicators were statistically independent and evaluated as individual risk factors to identify their specific associations with nocturia. While most studies on the relationship between nocturia and frailty have shown an association between nocturia and frailty, some have also shown negative results [12]. Our findings support the possibility of an association between nocturia and frailty. Therefore, managing frailty could be associated with better control of nocturia. Although our findings show a significant association, the bidirectional nature of this relationship must be considered. While frailty may contribute to nocturia, it is also possible that nocturia‐induced sleep fragmentation accelerates the progression of frailty.

Independent factors for nocturia identified in only men included depression/anxiety, BPH, fecal incontinence, alcohol intake, and ED. Although BPH and ED, which are highly prevalent in aging men, are associated with nocturia, their causal relationships remain unclear [13, 14]. Metabolic syndrome might play a key role in these disorders [13]. The identification of HT, a metabolic syndrome‐related disease, as an independent factor for nocturia in the present study is consistent with the potential shared associations of metabolic syndrome for BPH, ED, and nocturia.

In accordance with depression/anxiety, previous studies showed that a bidirectional relationship between nocturia and depression in men [15, 16]. Furthermore, in men 40 years old or older, a cross‐sectional study showed a significant association between LUTS including nocturia and depression [17]. Considering our results, which showed that nocturia was also prevalent in younger men and depression/anxiety was associated with nocturia in those aged < 60 years from subgroup analysis, we speculate that the associations between nocturia and depression may be stronger in relatively younger men.

Although one survey of geriatric rehabilitation inpatients showed an association between nocturia and fecal incontinence, this association has not been well examined [18]. Considering our result, the use of diapers for fecal incontinence may have been misinterpreted as nocturia.

Alcohol intake is considered to disrupt sleep and increase night‐time urine production, which may be related to nocturia. According to the National Health and Nutrition Survey in Japan, 2023 (https://www.mhlw.go.jp/stf/seisakunitsuite/bunya/kenkou_iryou/kenkou/eiyou/r5‐houkoku_00001.html), alcohol consumption rates are higher among men than among women. We speculate that our result reflects this trend.

Compared with men, decreased walking speed was identified as a significant factor only in women. Walking speed is an indicator of physical performance and its decline has been associated with frailty and nocturia [19, 20]. In Japan, the prevalence of frailty increases with age, and the rate is higher in women than in men aged over 65 years [21]. Thus, our findings suggest a stronger association between nocturia and frailty‐related indicators in women than in men. This indicates that addressing physical decline might be a potential strategy for managing nocturia, particularly in women.

Subgroup analyses suggested that factors associated with nocturia differed between individuals aged < 60 years and ≥ 60 years. Although nocturia increased with age, it was also observed in younger participants. In men aged < 60 years, HL and SAS were associated with nocturia. HL, a component of metabolic syndrome, has been associated with overactive bladder (OAB) [22]. The association between HL and nocturia may be related to the close relationship between OAB and nocturia.

SAS is more common in men, and nocturia is a common LUTS in patients with SAS. A systematic review showed that approximately one‐third of patients with SAS experienced nocturia, with a mean age of 53.3 years, and one third of studies included middle‐aged patients [23]. Moreover, a previous study showed no relationship between SAS and nocturia in elderly patients aged > 65 years with SAS [24]. Our result may reflect a higher prevalence of SAS among middle‐aged men.

Weight loss was identified as a factor associated with nocturia only in women aged < 60 years. In overweight or obese women, weight loss has been considered to positively affect LUTS, including nocturia [25]. Although our findings differ from previous reports, we speculate that this discrepancy may be because our participants were not exclusively overweight or obese. Furthermore, we could not conduct a detailed survey of daily food and dietary habits, which were important for body weight. To clarify the effectiveness of weight gain, weight loss, and dietary intake for nocturia, further research will be needed in the future.

POP commonly occurs in aged women and has been linked to OAB, including nocturia. Thus, our findings suggest an association between POP and nocturia in women aged ≥ 60 years.

This study has several limitations. First, while including participants in their 80s and 90s is a notable strength, the online format may have introduced selection bias by limiting participation to those with digital access. Additionally, although the sample was designed to match the 2020 National Census, post‐stratification weights were not applied. However, given the widespread use of smartphones and tablets across all age groups in Japan, we consider this method appropriate, though the potential for bias remains an inherent limitation. Second, the subgroup analyses were exploratory in nature. Given the potential for multiplicity, these results should be interpreted as hypothesis‐generating and further studies are necessary to confirm these findings. Third, because JaCS 2023 was not specifically designed for this study, we relied on self‐reported questionnaires regarding current treatment for comorbidities and lacked clinical data such as frequency–volume charts. This may involve misclassification or recall bias, and we could not definitively determine the specific etiology of nocturia. Fourth, our covariate selection was primarily based on univariable significance (*p* < 0.05). Although clinically key factors such as age and sex were accounted for, this approach may have omitted other potential confounders that did not reach statistical significance in the univariable analysis. Finally, owing to the cross‐sectional design, our findings reflect associations rather than causality, and the results may represent both the causes and consequences of nocturia. Nevertheless, we believe that the findings of this study provide timely and valuable insights for developing strategies to manage nocturia in a super‐aging society.

In conclusion, this study showed that the prevalence of nocturia increased with age and was more common in men than in women across all age groups in Japan. Age, PS ≥ 1, HT, and insomnia were identified as factors associated with nocturia in both men and women across all age groups. In a super aging society, these latest findings may contribute to a better understanding of the factors associated with nocturia.

## Author Contributions

**Satoru Kira:** conceptualization, methodology, writing – original draft, writing – review and editing, investigation, validation, formal analysis, visualization, data curation. **Hiroshi Shimura:** writing – review and editing. **Takanori Mochizuki:** writing – review and editing. **Norifumi Sawada:** writing – review and editing. **Noritoshi Sekido:** conceptualization, methodology, funding acquisition, investigation, project administration, validation. **Naoya Masumori:** conceptualization, funding acquisition, investigation, methodology, validation, project administration. **Yoshiyuki Kojima:** conceptualization, investigation, funding acquisition, methodology, validation, project administration. **Takahiko Mitsui:** conceptualization, methodology, funding acquisition, writing – review and editing, investigation, project administration, validation, data curation.

## Funding

This study was supported by the 50th Anniversary Project of the Japanese Continence Society.

## Ethics Statement

The protocol for this research project was approved by a suitably constituted institutional ethics committee (Nihon University Itabashi Hospital Clinical Research Judging Committee, ID 2023–04) and conformed to the provisions of the Declaration of Helsinki.

## Consent

The participants provided consent through the opt‐in consent method.

## Conflicts of Interest

Naoya Masumori is the Editor‐in‐Chie of International Journal of Urology and a co‐author of this article. Yoshiyuki Kojima, and Takahiko Mitsui are the Editorial Board members of the International Journal of Urology, and the co‐authors of this article.

Norifumi Sawada is the Deputy Editors of the International Journal of Urology, and a co‐author of this article. They were excluded from all editorial decision‐making related to the acceptance of this article for publication to minimize bias.

## Supporting information


**Table S1:** Participant characteristics between the Nocturia and non‐Nocturia groups in men aged < 60 years. Abbreviations: N, number; IQR, interquartile range; BMI, body mass index; PS, performance status; BPH, benign prostatic hyperplasia.
**Table S2:** Participant characteristics between the Nocturia and non‐Nocturia groups in men aged ≥ 60 years. Abbreviations: N, number; IQR, interquartile range; BMI, body mass index; PS, performance status; BPH, benign prostatic hyperplasia.
**Table S3:** Participant characteristics between the Nocturia and non‐Nocturia groups in women aged < 60 years. Abbreviations: N, number; IQR, interquartile range; BMI, body mass index; PS, performance status.
**Table S4:** Participant characteristics between the Nocturia and non‐Nocturia groups in women aged ≥ 60 years. Abbreviations: N, number; IQR, interquartile range; BMI, body mass index; PS, performance status.
**Table S5:** Subgroup analysis for nocturia in men aged < 60 years. Abbreviations: BMI, body mass index; PS, performance status; BPH, benign prostatic hyperplasia.
**Table S6:** Subgroup analysis for nocturia in men aged ≥ 60 years. Abbreviations: BMI, body mass index; PS, performance status; BPH, benign prostatic hyperplasia.
**Table S7:** Subgroup analysis for nocturia in women aged < 60 years. Abbreviation: BMI, body mass index; PS, performance status.
**Table S8:** Subgroup analysis for nocturia in women aged ≥ 60 years. Abbreviation: BMI, body mass index; PS, performance status.

## Data Availability

The data that support the findings of this study are available from the Japanese Continence Society. Restrictions apply to the availability of these data, which were used under license for this study. Data are available from the author(s) with the permission of the Japanese Continence Society.
