# Supplementary material for: Prevalence and Factors Associated With Nocturia (≥ 2 Night‐Time Urinary Episodes) in Japan: Analysis of the 2023 Japan Community Health Survey (JaCS 2023)
Source: Int J Urol. 2026 May 15;33:e70504. doi: 10.1111/iju.70504 (PMC13179475; doi:10.1111/iju.70504)
Supplement: Supplementary file 1 — Table S1: Participant characteristics between the Nocturia and non‐Nocturia groups in men aged < 60 years. Abbreviations: N, number; IQR, interquartile range; BMI, body mass index; PS, performance status; BPH, benign prostatic hyperplasia. Table S2: Participant characteristics between the Nocturia and non‐Nocturia groups in men aged ≥ 60 years. Abbreviations: N, number; IQR, interquartile range; BMI, body mass index; PS, performance status; BPH, benign prostatic hyperplasia. Table S3: Participant characteristics between the Nocturia and non‐Nocturia groups in women aged < 60 years. Abbreviations: N, number; IQR, interquartile range; BMI, body mass index; PS, performance status. Table S4: Participant characteristics between the Nocturia and non‐Nocturia groups in women aged ≥ 60 years. Abbreviations: N, number; IQR, interquartile range; BMI, body mass index; PS, performance status. Table S5: Subgroup analysis for nocturia in men aged < 60 years. Abbreviations: BMI, body mass index; PS, performance status; BPH, benign prostatic hyperplasia. Table S6: Subgroup analysis for nocturia in men aged ≥ 60 years. Abbreviations: BMI, body mass index; PS, performance status; BPH, benign prostatic hyperplasia. Table S7: Subgroup analysis for nocturia in women aged < 60 years. Abbreviation: BMI, body mass index; PS, performance status. Table S8: Subgroup analysis for nocturia in women aged ≥ 60 years. Abbreviation: BMI, body mass index; PS, performance status. [file IJU-33-0-s001.docx]

**Supporting Information**

Table S1. Participant characteristics between the Nocturia and non-Nocturia groups in men aged < 60 years

|  | **Non-nocturia** | **Nocturia** | ***p*-value** |
| --- | --- | --- | --- |
| N | 1549 | 367 |  |
| Age, median (IQR) | 42 (32-50) | 46 (36-52) | < 0.0001 |
| BMI, median (IQR) | 22.65 (20.66-25.53) | 23.03 (21.22-26.03) | 0.0347 |
| PS ≧1 | 125 (8.07) | 79 (21.53) | < 0.0001 |
| Relatively poor or poor health condition | 164 (10.59) | 77 (20.98) | < 0.0001 |
| Weight loss | 400 (25.82) | 126 (34.33) | 0.001 |
| Decreased walking speed | 345 (22.27) | 130 (35.42) | < 0.0001 |
| Fall | 301 (19.43) | 110 (29.97) | < 0.0001 |
| Lack of exercise | 730 (47.13) | 153 (41.69) | 0.0602 |
| Hypertension | 186 (12.01) | 79 (21.53) | < 0.0001 |
| Hyperlipidemia | 114 (7.36) | 41 (11.17) | 0.016 |
| Diabetes mellitus | 71 (4.58) | 46 (12.53) | < 0.0001 |
| Heart failure | 30 (1.94) | 17 (4.63) | 0.0027 |
| Myocardial infarction  Myocardial Infarction | 30 (1.94) | 20 (5.45) | 0.0001 |
| Chronic kidney disease | 24 (1.55) | 21 (5.72) | < 0.0001 |
| Stroke | 23 (1.48) | 16 (4.36) | 0.0005 |
| Spine/Spinal cord disorder | 18 (1.16) | 17 (4.63) | < 0.0001 |
| Neurologic disease | 21 (1.36) | 19 (5.18) | < 0.0001 |
| Depression/Anxiety | 106 (6.84) | 71 (19.35) | < 0.0001 |
| Sleep apnea syndrome | 48 (3.10) | 44 (11.99) | < 0.0001 |
| Insomnia | 81 (5.23) | 68 (18.53) | < 0.0001 |
| BPH | 30 (1.94) | 30 (8.17) | < 0.0001 |
| Fecal incontinence | 17 (1.10) | 24 (6.54) | < 0.0001 |
| Constipation | 93 (6.00) | 56 (15.26) | < 0.0001 |
| Alcohol intake | 699 (45.13) | 208 (56.68) | < 0.0001 |
| Erectile dysfunction | 186 (12.01) | 80 (21.80) | < 0.0001 |

Abbreviations: N, number; IQR, interquartile range; BMI, body mass index; PS, performance status; BPH, benign prostatic hyperplasia

Table S2. Participant characteristics between the Nocturia and non-Nocturia groups in men aged ≥ 60 years

|  | **Non-nocturia** | **Nocturia** | ***p*-value** |
| --- | --- | --- | --- |
| N | 726 | 480 |  |
| Age, median (IQR) | 70 (64-75) | 73 (67-81) | < 0.0001 |
| BMI, median (IQR) | 22.99 (21.09-24.96) | 22.97 (21.38-24.85) | 0.6472 |
| PS ≧1 | 129 (17.77) | 136 (28.33) | < 0.0001 |
| Relatively poor or poor health condition | 82 (11.29) | 85 (17.71) | 0.0016 |
| Weight loss | 118 (16.25) | 93 (19.38) | 0.1625 |
| Decreased walking speed | 340 (46.83) | 283 (58.96) | < 0.0001 |
| Fall | 131 (18.04) | 119 (24.79) | 0.0047 |
| Lack of exercise | 295 (40.63) | 189 (39.38) | 0.6625 |
| Hypertension | 288 (39.67) | 243 (50.63) | 0.0002 |
| Hyperlipidemia | 132 (18.18) | 96 (20.00) | 0.4299 |
| Diabetes mellitus | 118 (16.25) | 90 (18.75) | 0.2613 |
| Heart failure | 15 (2.07) | 24 (5.00) | 0.0048 |
| Myocardial infarction  Myocardial Infarction | 49 (6.75) | 33 (6.88) | 0.9324 |
| Chronic kidney disease | 22 (3.03) | 19 (3.96) | 0.384 |
| Stroke | 33 (4.55) | 23 (4.79) | 0.8423 |
| Spine/Spinal cord disorder | 21 (2.89) | 28 (5.83) | 0.0113 |
| Neurologic disease | 10 (1.38) | 11 (2.29) | 0.2348 |
| Depression/Anxiety | 14 (1.93) | 13 (2.71) | 0.3702 |
| Sleep apnea syndrome | 31 (4.27) | 29 (6.04) | 0.166 |
| Insomnia | 35 (4.82) | 38 (7.92) | 0.0273 |
| BPH | 73 (10.06) | 101 (21.04) | < 0.0001 |
| Fecal incontinence | 12 (1.65) | 32 (6.67) | < 0.0001 |
| Constipation | 53 (7.30) | 68 (14.17) | 0.0001 |
| Alcohol intake | 395 (54.41) | 288 (60.00) | 0.0551 |
| Erectile dysfunction | 259 (35.67) | 238 (49.58) | < 0.0001 |

Abbreviations: N, number; IQR, interquartile range; BMI, body mass index; PS, performance status; BPH, benign prostatic hyperplasia

Table S3. Participant characteristics between the Nocturia and non-Nocturia groups in women aged < 60 years

|  | **Non-nocturia** | **Nocturia** | ***p*-value** |
| --- | --- | --- | --- |
| N | 1517 | 217 |  |
| Age, median (IQR) | 42 (32-50) | 43 (34-50) | 0.2819 |
| BMI, median (IQR) | 20.58 (18.80-22.82) | 20.40 (18.86-23.76) | 0.5754 |
| PS ≧1 | 90 (5.93) | 33 (15.21) | < 0.0001 |
| Relatively poor or poor health condition | 126 (8.31) | 34 (15.67) | 0.0005 |
| Weight loss | 353 (23.27) | 71 (32.72) | 0.0025 |
| Decreased walking speed | 430 (28.35) | 88 (40.55) | 0.0002 |
| Fall | 281 (18.52) | 53 (24.42) | 0.0392 |
| Lack of exercise | 890 (58.67) | 133 (61.29) | 0.4626 |
| Hypertension | 77 (5.08) | 21 (9.68) | 0.006 |
| Hyperlipidemia | 80 (5.27) | 17 (7.83) | 0.1247 |
| Diabetes mellitus | 35 (2.31) | 7 (3.23) | 0.4103 |
| Heart failure | 18 (1.19) | 7 (3.23) | 0.0184 |
| Myocardial infarction | 16 (1.05) | 5 (2.30) | 0.1155 |
| Chronic kidney disease | 23 (1.52) | 8 (3.69) | 0.024 |
| Stroke | 17 (1.12) | 6 (2.76) | 0.0477 |
| Spine/Spinal cord disorder | 18 (1.19) | 7 (3.23) | 0.0184 |
| Neurologic disease | 24 (1.58) | 4 (1.84) | 0.7752 |
| Depression/Anxiety | 124 (8.17) | 21 (9.68) | 0.4543 |
| Sleep apnea syndrome | 27 (1.78) | 6 (2.76) | 0.3205 |
| Insomnia | 96 (6.33) | 34 (15.67) | < 0.0001 |
| Fecal incontinence | 16 (1.05) | 1 (0.46) | 0.4063 |
| Constipation | 190 (12.52) | 36 (16.59) | 0.0962 |
| Alcohol intake | 436 (28.74) | 71 (32.72) | 0.2282 |
| Parity |  |  | 0.4581 |
| 1 ~ 2 | 612 (40.34) | 84 (38.71) |  |
| ≧3 | 113 (7.45) | 19 (8.76) |  |
| Menopause | 284 (18.72) | 38 (17.51) | 0.7536 |
| Pelvic organ prolapse | 84 (5.54) | 14 (6.45) | 0.503 |

Abbreviations: N, number; IQR, interquartile range; BMI, body mass index; PS, performance status.

Table S4. Participant characteristics between the Nocturia and non-Nocturia groups in women aged ≥ 60 years

|  | **Non-nocturia** | **Nocturia** | ***p*-value** |
| --- | --- | --- | --- |
| N | 1020 | 334 |  |
| Age, median (IQR) | 71 (65-78) | 76 (70.75-82.25) | < 0.0001 |
| BMI, median (IQR) | 21.22 (19.30-23.49) | 23.03 (21.22-26.03) | 0.2781 |
| PS ≧1 | 174 (17.06) | 142 (42.51) | < 0.0001 |
| Relatively poor or poor health condition | 100 (9.80) | 83 (24.85) | < 0.0001 |
| Weight loss | 145 (14.22) | 60 (17.96) | 0.0972 |
| Decreased walking speed | 433 (42.45) | 216 (64.67) | < 0.0001 |
| Fall | 210 (20.59) | 98 (29.34) | 0.0009 |
| Lack of exercise | 409 (40.10) | 148 (44.31) | 0.1744 |
| Hypertension | 297 (29.12) | 147 (44.01) | < 0.0001 |
| Hyperlipidemia | 193 (18.92) | 79 (23.65) | 0.0611 |
| Diabetes mellitus | 63 (6.18) | 37 (11.08) | 0.003 |
| Heart failure | 11 (1.08) | 18 (5.39) | < 0.0001 |
| Myocardial infarction | 23 (2.25) | 21 (6.29) | 0.0003 |
| Chronic kidney disease | 12 (1.18) | 14 (4.19) | 0.0005 |
| Stroke | 19 (1.86) | 16 (4.79) | 0.0034 |
| Spine/Spinal cord disorder | 35 (3.43) | 27 (8.08) | 0.0004 |
| Neurologic disease | 7 (0.69) | 11 (3.29) | 0.0003 |
| Depression/Anxiety | 30 (2.94) | 23 (6.89) | 0.0013 |
| Sleep apnea syndrome | 11 (1.08) | 13 (3.89) | 0.0007 |
| Insomnia | 62 (6.08) | 38 (11.38) | 0.0013 |
| Fecal incontinence | 20 (1.96) | 34 (10.18) | < 0.0001 |
| Constipation | 141 (13.82) | 67 (20.06) | 0.0061 |
| Alcohol intake | 303 (29.71) | 93 (27.84) | 0.5163 |
| Parity |  |  | 0.4154 |
| 1 ~ 2 | 615 (60.29) | 201 (60.18) |  |
| ≧3 | 199 (19.51) | 74 (22.16) | 0.4341 |
| Menopause | 802 (78.63) | 242 (72.46) | 0.0002 |
| Pelvic organ prolapse | 23 (2.25) | 21 (6.29) | 0.4154 |

Abbreviations: N, number; IQR, interquartile range; BMI, body mass index; PS, performance status.

Table S5. Subgroup analyses for nocturia in men aged < 60 years

|  | **Univariable analysis** | | | **Multivariable analysis** | | | |  |
| --- | --- | --- | --- | --- | --- | --- | --- | --- |
|  | Odds ratio | 95% CI | *p*-value | | Odds ratio | 95% CI | *p*-value | |
| Age | 1.023 | 1.012-1.034 | < 0.0001 | | 1.022 | 1.008-1.035 | 0.0016 | |
| BMI | 1.022 | 0.994-1.050 | 0.1194 | |  |  |  | |
| PS ≧1 | 3.125 | 2.295-4.256 | < 0.0001 | | 2.101 | 1.385-3.188 | 0.0005 | |
| Relatively poor or poor health condition | 2.242 | 1.663-3.023 | < 0.0001 | | 1.219 | 0.828-1.796 | 0.3159 | |
| Weight loss | 1.502 | 1.177-1.916 | 0.0011 | | 1.01 | 0.744-1.372 | 0.9478 | |
| Decreased walking speed | 1.914 | 1.498-2.446 | < 0.0001 | | 1.133 | 0.818-1.568 | 0.4523 | |
| Fall | 1.775 | 1.373-2.293 | < 0.0001 | | 1.17 | 0.845-1.622 | 0.3447 | |
| Lack of Exercise | 1.247 | 0.990-1.569 | 0.0605 | |  |  |  | |
| Hypertension | 2.01 | 1.501-2.693 | < 0.0001 | | 1.225 | 0.836-1.795 | 0.2984 | |
| Hyperlipidemia | 1.583 | 1.086-2.307 | 0.0168 | | 0.558 | 0.327-0.952 | 0.0323 | |
| Diabetes mellitus | 2.983 | 2.020-4.406 | < 0.0001 | | 1.411 | 0.813-2.449 | 0.2215 | |
| Heart failure | 2.459 | 1.341-4.509 | 0.0036 | | 0.363 | 0.114-1.154 | 0.0861 | |
| Myocardial infarction | 2.918 | 1.638-5.200 | 0.0003 | | 0.848 | 0.285-2.525 | 0.7676 | |
| Chronic kidney disease | 3.857 | 2.123-7.007 | < 0.0001 | | 1.471 | 0.582-3.714 | 0.4144 | |
| Stroke | 3.024 | 1.581-5.785 | 0.0008 | | 0.596 | 0.165-2.160 | 0.431 | |
| Spine/Spinal cord disorder | 4.131 | 2.108-8.097 | < 0.0001 | | 1.877 | 0.641-5.495 | 0.251 | |
| Neurologic disease | 3.973 | 2.113-7.469 | < 0.0001 | | 0.884 | 0.259-3.009 | 0.8432 | |
| Depression/Anxiety | 3.265 | 2.358-4.522 | < 0.0001 | | 1.65 | 1.056-2.580 | 0.028 | |
| Sleep Apnea Syndrome | 4.26 | 2.781-6.525 | < 0.0001 | | 1.943 | 1.089-3.467 | 0.0246 | |
| Insomnia | 4.122 | 2.918-5.823 | < 0.0001 | | 1.624 | 0.991-2.663 | 0.0544 | |
| BPH | 4.507 | 2.681-7.579 | < 0.0001 | | 1.627 | 0.793-3.340 | 0.1847 | |
| Fecal Incontinence | 6.306 | 3.351-11.866 | < 0.0001 | | 2.223 | 0.891-5.543 | 0.0867 | |
| Constipation | 2.819 | 1.980-4.014 | < 0.0001 | | 1.051 | 0.651-1.697 | 0.839 | |
| Alcohol intake | 1.591 | 1.265-2.001 | < 0.0001 | | 1.561 | 1.190-2.048 | 0.0013 | |
| Erectile Dysfunction | 2.079 | 1.543-2.802 | < 0.0001 | | 1.453 | 1.040-2.031 | 0.0285 | |

; *p*<0.05

Abbreviations: BMI, body mass index; PS, performance status; BPH, benign prostatic hyperplasia

Table S6. Subgroup analyses for nocturia in men aged ≥ 60 years

|  | **Univariable analysis** | | | **Multivariable analysis** | | | |  |
| --- | --- | --- | --- | --- | --- | --- | --- | --- |
|  | Odds ratio | 95% CI | *p*-value | | Odds ratio | 95% CI | *p*-value | |
| Age | 1.056 | 1.041-1.072 | < 0.0001 | | 1.206 | 1.020-1.059 | < 0.0001 | |
| BMI | 0.993 | 0.964-1.007 | 0.5031 | |  |  |  | |
| PS ≧1 | 1.83 | 1.390-2.409 | < 0.0001 | | 0.955 | 0.657-1.388 | 0.8099 | |
| Relatively poor or poor health condition | 1.69 | 1.217-2.347 | 0.0017 | | 1.312 | 0.858-2.005 | 0.21 | |
| Weight loss | 1.238 | 0.917-1.672 | 0.163 | |  |  |  | |
| Decreased walking speed | 1.631 | 1.292-2.059 | < 0.0001 | | 1.041 | 0.774-1.401 | 0.7896 | |
| Fall | 1.497 | 1.131-1.982 | 0.0048 | | 1.219 | 0.880-1.689 | 0.2339 | |
| Lack of exercise | 1.053 | 0.833-1.334 | 0.6625 | |  |  |  | |
| Hypertension | 1.559 | 1.236-1.968 | 0.0002 | | 1.375 | 1.051-1.801 | 0.0206 | |
| Hyperlipidemia | 1.125 | 0.840-1.507 | 0.4301 | |  |  |  | |
| Diabetes mellitus | 1.189 | 0.879-1.609 | 0.2616 | |  |  |  | |
| Heart failure | 2.495 | 1.295-4.806 | 0.0063 | | 1.151 | 0.550-2.411 | 0.7089 | |
| Myocardial infarction | 1.02 | 0.646-1.611 | 0.9324 | |  |  |  | |
| Chronic Kidney Disease | 1.319 | 0.706-2.464 | 0.3854 | |  |  |  | |
| Stroke | 1.057 | 0.613-1.823 | 0.8424 | |  |  |  | |
| Spine/Spinal cord disorder | 2.08 | 1.167-3.707 | 0.013 | | 1.093 | 0.544-2.193 | 0.8031 | |
| Neurologic disease | 1.679 | 0.708-3.985 | 0.2397 | |  |  |  | |
| Depression/Anxiety | 1.416 | 0.660-3.039 | 0.3724 | |  |  |  | |
| Sleep apnea syndrome | 1.442 | 0.857-2.424 | 0.168 | |  |  |  | |
| Insomnia | 1.697 | 1.056-2.728 | 0.0288 | | 1.359 | 0.758-2.434 | 0.3029 | |
| BPH | 2.384 | 1.720-3.305 | < 0.0001 | | 1.6 | 1.096-2.335 | 0.015 | |
| Fecal incontinence | 4.25 | 2.166-8.338 | < 0.0001 | | 2.144 | 0.994-4.627 | 0.0519 | |
| Constipation | 2.096 | 1.434-3.063 | 0.0001 | | 1.34 | 0.856-2.098 | 0.2001 | |
| Alcohol intake | 1.257 | 0.995-1.588 | 0.0552 | |  |  |  | |
| Erectile Dysfunction | 2.022 | 1.567-2.608 | < 0.0001 | | 1.412 | 1.063-1.875 | 0.0173 | |

; *p*<0.05

Abbreviations: BMI, body mass index; PS, performance status; BPH, benign prostatic hyperplasia

Table S7. Subgroup analyses for nocturia in women aged < 60 years

|  | **Univariable analysis** | | | **Multivariable analysis** | | |
| --- | --- | --- | --- | --- | --- | --- |
|  | Odds ratio | 95% CI | *p*-value | Odds ratio | 95% CI | *p*-value |
| Age | 1.008 | 0.995-1.022 | 0.2188 | 1.01 | 0.996-1.025 | 0.1592 |
| BMI | 1.013 | 1.001-1.038 | 0.1844 |  |  |  |
| PS≧1 | 2.844 | 1.855-4.360 | < 0.0001 | 1.925 | 1.186-3.126 | 0.0081 |
| Relatively poor or poor health condition | 2.051 | 1.363-3.087 | 0.0006 | 1.203 | 0.751-1.926 | 0.4415 |
| Weight loss | 1.604 | 1.179-2.181 | 0.0026 | 1.41 | 1.017-1.955 | 0.0395 |
| Decreased walking speed | 1.724 | 1.286-2.312 | 0.0003 | 1.298 | 0.943-1.788 | 0.11 |
| Fall | 1.421 | 1.016-1.989 | 0.04 | 1.102 | 0.771-1.575 | 0.5952 |
| Lack of Exercise | 0.896 | 0.670-1.200 | 0.4628 |  |  |  |
| Hypertension | 2.004 | 1.209-3.320 | 0.007 | 1.26 | 0.694-2.288 | 0.4466 |
| Hyperlipidemia | 1.527 | 0.886-2.630 | 0.1273 |  |  |  |
| Diabetes mellitus | 1.411 | 0.619-3.218 | 0.4126 |  |  |  |
| Heart failure | 2.776 | 1.146-6.725 | 0.0237 | 0.937 | 0.257-3.414 | 0.9218 |
| Myocardial infarction | 2.213 | 0.802-6.102 | 0.1249 |  |  |  |
| Chronic kidney disease | 2.486 | 1.098-5.631 | 0.029 | 1.106 | 0.357-3.434 | 0.8608 |
| Stroke | 2.509 | 0.978-6.435 | 0.0556 |  |  |  |
| Spine/Spinal cord disorder | 2.776 | 1.146-6.725 | 0.0237 | 1.508 | 0.479-4.751 | 0.4831 |
| Neurologic disease | 1.168 | 0.401-3.400 | 0.7754 |  |  |  |
| Depression/Anxiety | 1.204 | 0.740-1.957 | 0.4549 |  |  |  |
| Sleep apnea syndrome | 1.569 | 0.640-3.845 | 0.3244 |  |  |  |
| Insomnia | 2.75 | 1.806-4.187 | < 0.0001 | 1.884 | 1.174-3.023 | 0.0087 |
| Fecal incontinence | 0.434 | 0.057-3.291 | 0.4196 |  |  |  |
| Constipation | 1.389 | 0.942-2.049 | 0.0974 |  |  |  |
| Alcohol intake | 1.206 | 0.889-1.635 | 0.2287 |  |  |  |
| Parity ≧3 | 1.225 | 0.699-2.056 | 0.4657 |  |  |  |
| Menopause | 0.942 | 0.646-1.372 | 0.7537 |  |  |  |
| Pelvic organ prolapse | 1.222 | 0.679-2.196 | 0.5036 |  |  |  |

; *p*<0.05

Abbreviation: BMI, body mass index; PS, performance status

Table S8. Subgroup analyses for nocturia in women aged ≥ 60 years

|  | **Univariable analysis** | | | **Multivariable analysis** | | |
| --- | --- | --- | --- | --- | --- | --- |
|  | Odds ratio | 95% CI | *p*-value | Odds ratio | 95% CI | *p*-value |
| Age | 1.061 | 1.045-1.076 | < 0.0001 | 1.033 | 1.015-1.052 | 0.0003 |
| BMI | 1.021 | 0.988-1.054 | 0.2111 |  |  |  |
| PS ≧1 | 3.596 | 2.741-4.717 | < 0.0001 | 1.909 | 1.322-2.756 | 0.0006 |
| Relatively poor or poor health condition | 3.042 | 2.203-4.201 | < 0.0001 | 1.359 | 0.892-2.068 | 0.1529 |
| Weight loss | 1.321 | 0.950-1.838 | 0.0979 |  |  |  |
| Decreased walking speed | 2.482 | 1.920-3.207 | < 0.0001 | 1.449 | 1.058-1.986 | 0.021 |
| Fall | 1.602 | 1.210-2.120 | 0.001 | 0.96 | 0.684-1.346 | 0.8116 |
| Lack of Exercise | 0.841 | 0.655-1.080 | 0.1747 |  |  |  |
| Hypertension | 1.914 | 1.483-2.469 | < 0.0001 | 1.348 | 1.003-1.811 | 0.0475 |
| Hyperlipidemia | 1.328 | 0.986-1.787 | 0.0616 |  |  |  |
| Diabetes mellitus | 1.892 | 1.236-2.898 | 0.0034 | 0.853 | 0.496-1.469 | 0.5675 |
| Heart Failure | 5.225 | 2.442-11.180 | < 0.0001 | 1.517 | 0.532-4.322 | 0.4357 |
| Myocardial infarction | 2.908 | 1.588-5.326 | 0.0005 | 1.213 | 0.552-2.665 | 0.6314 |
| Chronic Kidney Disease | 3.675 | 1.683-8.027 | 0.0011 | 1.612 | 0.541-4.801 | 0.3914 |
| Stroke | 2.651 | 1.347-5.216 | 0.0048 | 1.011 | 0.416-2.459 | 0.9799 |
| Spine/Spinal cord disorder | 2.475 | 1.474-4.155 | 0.0006 | 0.913 | 0.480-1.738 | 0.7821 |
| Neurologic disease | 4.928 | 1.895-12.818 | 0.0011 | 0.455 | 0.105-1.960 | 0.2903 |
| Depression/Anxiety | 2.441 | 1.397-4.264 | 0.0017 | 1.805 | 0.869-3.750 | 0.1136 |
| Sleep apnea syndrome | 3.715 | 1.648-8.373 | 0.0016 | 1.399 | 0.478-4.096 | 0.5402 |
| Insomnia | 1.984 | 1.298-3.032 | 0.0016 | 1.31 | 0.756-2.268 | 0.3356 |
| Fecal Incontinence | 5.667 | 3.214-9.992 | < 0.0001 | 2.328 | 1.157-4.682 | 0.0178 |
| Constipation | 1.564 | 1.134-2.157 | 0.0064 | 0.99 | 0.673-1.455 | 0.9576 |
| Alcohol intake | 0.913 | 0.694-1.201 | 0.5163 |  |  |  |
| Parity ≧3 | 1.138 | 0.834-1.552 | 0.4156 |  |  |  |
| Menopause | 0.717 | 0.310-1.657 | 0.4361 |  |  |  |
| Pelvic organ prolapse | 3.018 | 1.646-5.534 | 0.0004 | 2.079 | 1.045-4.136 | 0.0369 |

; *p*<0.05

Abbreviation: BMI, body mass index; PS, performance status
